# Thermal and Mechanical Behavior of Polyimide–Polyurea Copolymers: Insights from Molecular Dynamics Simulations

**DOI:** 10.3390/polym18141779

**Published:** 2026-07-21

**Authors:** Shuaijiang Ma, Yizi Chen, Desen Cheng, Dongwei Xu, Xuyan Li, Baocheng Yang, Shiwei Wang

**Affiliations:** 1 Zhengzhou Key Laboratory of Fiber Reinforced Polymer Composites, Faculty of Engineering, Huanghe S&T University, Zhengzhou 450063, China; cds@hhstu.edu.cn (D.C.); lxy@hhstu.edu.cn (X.L.);; 2 High-Tech Organic Fibers and Composite Materials Key Laboratory of Sichuan Province, China Bluestar Chengrand Co., Ltd., Chengdu 610041, China; chenyizi0526@foxmail.com; 3 Henan Key Laboratory of Aeronautical Materials and Application Technology, School of Materials Science and Engineering, Zhengzhou University of Aeronautics, Zhengzhou 450046, China; xudongwei1029@126.com; 4 National Center for International Joint Research of Micro-Nano Molding Technology, School of Mechanics and Safety Engineering, Zhengzhou University, Zhengzhou 450001, China

**Keywords:** all-atom molecular dynamics, thermal–mechanical properties, polyimide-polyurea copolymers, structure–property relationships

## Abstract

Polyimide (PI) exhibits outstanding thermal stability and mechanical rigidity; however, their inherently rigid backbones lead to intrinsic brittleness, poor fracture toughness, and inferior impact resistance. Conversely, polyurea (PUA) features excellent elasticity, tunable soft–hard segment architectures, and a favorable balance of tensile strength and elongation at break. Herein, we systematically investigate the thermal and mechanical properties of 12 distinct PI, PUA, and PI-PUA copolymer systems via all-atom molecular dynamics simulations. Simulations demonstrate that rigid aromatic moieties significantly increase *T_g_* and elastic modulus, while flexible hexamethylene diisocyanate (HDI) yields the highest elastic modulus via dense hydrogen-bond networks despite lowering *T_g_*. Fluorine substitution effectively increases fractional free volume and moderately reduces *T_g_*. Toughness is evaluated by *K/G*. System L with bulky phthalide side groups exhibits the highest *K/G* of 3.24, suggesting potential for improved plastic deformability as a preliminary screening indicator. In contrast, HDI-containing systems E and H show the lowest *K/G* ratios, as strong interchain hydrogen bonding severely restricts segmental slippage and induces brittle fracture. PI-PUA copolymerization proves to be an effective strategy to balance stiffness and toughness over a broad performance range. This work establishes structure–property correlations for PI-PUA systems, offering molecular-level insights for the rational design of advanced high-performance polymers, which require further experimental validation.

## 1. Introduction

Polyimide (PI) and polyurea (PUA) represent two distinct classes of high-performance polymers that have found extensive applications across diverse industrial sectors. Nevertheless, each material class suffers from inherent performance trade-offs when utilized in isolation. Most commercial high-performance PIs exhibit outstanding thermal stability and mechanical rigidity due to their highly rigid aromatic backbones; however, this inherent rigidity leads to intrinsic brittleness, poor fracture toughness, and inferior impact resistance [1,2]. In contrast, PUA exhibits comparatively lower mechanical strength and thermal stability; yet their abundant urea linkages form extensive hydrogen-bond networks that impart exceptional adhesive properties and dramatically improved fracture toughness [3,4].

In recent years, the copolymerization of PI and PUA segments has emerged as a promising strategy to reconcile the conflicting requirements of thermal stability and mechanical toughness, becoming a focal point of research in advanced polymeric material design [5,6]. Nguyen et al. [7] pioneered the introduction of urea linkages into polyimide backbones with low-cost monomers and successfully fabricated polyimide–urea (PIU) aerogels categorized as high-performance materials, which exhibit exceptional thermal stability with no aging observed after 500 h at 200 °C and outstanding chemical corrosion resistance. Song et al. [8] developed a series of high-performance poly(amide-urea)s (NIPURs) via melt polycondensation, achieving the simultaneous enhancement of yield strength and elongation at break while preserving high melting temperatures and thermal stability. Nicholls et al. [9] characterized the thermomechanical properties of thermoplastic PI-PUA copolymers, demonstrating that rigid imide rings and urea hydrogen bonds exert a synergistic effect in strengthening interchain interactions, thereby simultaneously enhancing the thermal stability and mechanical strength of the copolymers. Collectively, these studies establish that the introduction of urea linkages reinforces interchain hydrogen bonding in thermoplastic PI-PUA copolymers, which in turn improves matrix toughness and imparts self-healing capabilities [10,11,12,13,14].

Despite substantial advances in PI-PUA copolymer modification research, existing experimental investigations have predominantly focused on optimizing synthesis protocols and characterizing macroscopic properties. A systematic, in-depth understanding of the molecular mechanisms by which distinct chemical structural moieties govern the thermal and mechanical properties of these materials remains elusive. Molecular dynamics (MD) simulation, as a pivotal research approach bridging microscopic molecular architectures and macroscopic performance, has been extensively employed to explore structure–property relationships in high-performance polymers.

The regulatory effects of PI backbone rigidity and structural diversity on glass transition temperature, elastic modulus, and free volume characteristics have confirmed that molecular chain packing density and cohesive energy serve as the core microscopic factors governing the macroscopic performance of such materials [10]. Multiscale simulation investigations of PUA systems have elucidated the dynamic evolution behavior of hydrogen bond networks at the molecular level, verifying that hard-segment hydrogen bond density and the degree of microphase separation are critical determinants of material toughness and energy absorption capacity. Furthermore, both the isocyanate structure and soft segment ratio can tailor macroscopic properties by modifying hydrogen bond formation capability [15,16,17].

Several research teams have developed a variety of high-performance thermosetting resin systems by incorporating polar functional groups including cyano, phthalide, and fluorene moieties [18,19]. Phthalide-modified polymers typically combine favorable processability, excellent mechanical strength, and remarkable heat resistance [20]. The steric hindrance of naphthalene rings disrupts the ordered packing of molecular chains, increases the free volume fraction, and attenuates intermolecular forces, thus improving the moisture resistance of the materials. This effect offers a valuable design strategy for developing low-dielectric, high-heat-resistant polymer materials [21,22,23]. The introduction of fluorine atoms incorporates highly electronegative groups into the system, triggering intramolecular repulsion and generating additional free volume within the polymer matrix, which in turn weakens interchain interactions. This effect causes the glass transition temperature of the material to decrease regularly with increasing fluorine content [24]. The strong polarization effect of fluorine atoms can substantially modify dipole–dipole interactions between molecular chains, while regulating free volume distribution via steric hindrance to achieve synergistic optimization of dielectric and mechanical properties. This provides a theoretical foundation for the molecular design of fluorine-containing monomers [25]. Based on the analysis of the aforementioned MD simulation studies, most existing molecular simulation studies are largely restricted to single homopolymer systems or a limited number of specific copolymer compositions, lacking systematic comparisons of multiple key structural motifs across different polymer classes. This knowledge gap significantly hinders the rational design of high-performance PI-PUA materials with tailored properties.

To address these critical knowledge gaps, we have constructed all-atom models of 12 distinct PI homopolymer, PUA homopolymer, and PI-PUA copolymer systems with systematically varied molecular compositions. Extensive molecular dynamics simulations were then performed to systematically investigate the influence of six key structural motifs—benzene rings, fluorine substituents, cyclohexane rings, naphthalene rings, aliphatic chains, and phthalide side groups—on *T_g_*, elastic modulus, shear modulus, bulk modulus, toughness, fractional free volume (FFV), cohesive energy density (CED), and hydrogen-bond network characteristics. The overarching goal of this work is to establish structure–property correlations and provide molecular-level mechanisms and guiding future experimental synthesis, rather than providing directly applicable material designs.

## 2. Computational Methodology

**Figure 1:**
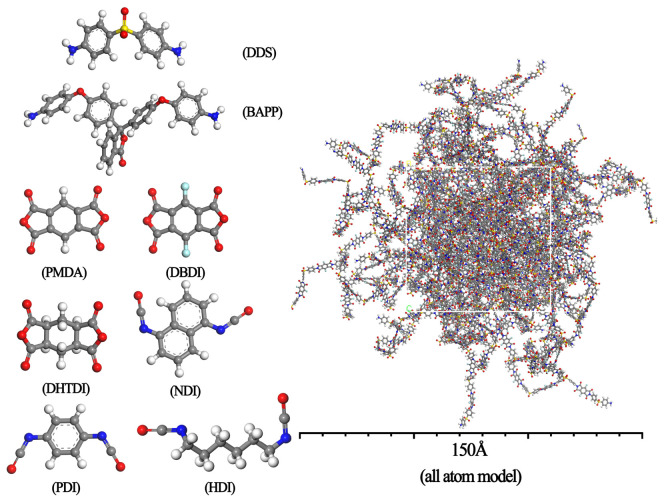
Ball-and-stick models of monomer and polymer all-atom models. Color legend: hydrogen H (white); nitrogen N (blue); carbon C (gray); oxygen O (red); sulfur S (yellow); fluorine F (cyan).

All monomer structures and all-atom models of PI, PUA, and PI-PUA were constructed using the Materials Studio 2020 software suite (BIOVIA, San Diego, CA, USA). The chemical structures of the monomers employed in this study are depicted in Figure 1. The monomers include: 4,4′-diaminodiphenyl sulfone (DDS), pyromellitic dianhydride (PMDA), 1,4-difluoro-2,3,5,6-benzenetetracarboxylic dianhydride (DBDI), 1,2,4,5-cyclohexanetetracarboxylic dianhydride (DHTDI), HDI, naphthalene diisocyanate (NDI), p-phenylene diisocyanate (PDI), and 3,3-bis(4-(4-aminophenoxy)phenyl)phthalide (BAPP). The phthalide moiety was incorporated into the diamine backbone to produce the chain-extended diamine BAPP (Figure 1).

**Figure 2:**
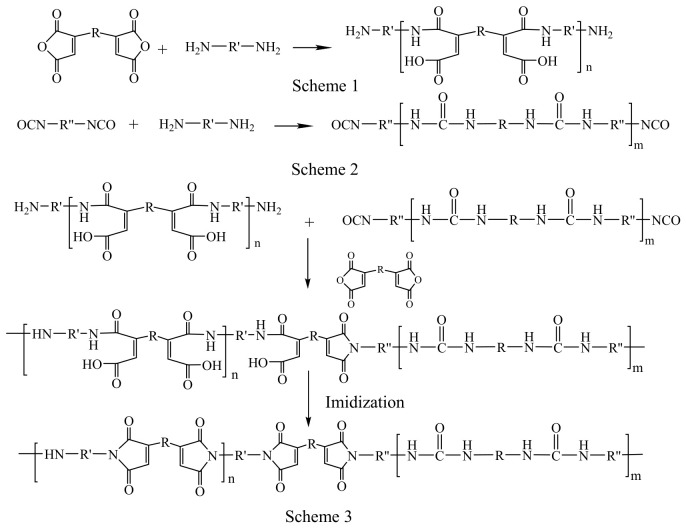
Schematic illustration of the construction of PI-PUA copolymers.

The general synthetic strategy for PI–PUA copolymers involves the initial reaction between diamines and dianhydrides to generate amine-terminated poly(amic acid) (PAA) precursors; these precursors are further reacted with diisocyanates or urea-containing isocyanate-terminated oligomers pre-synthesized by diamine–diisocyanate condensation, and the desired PI–PUA copolymers are finally prepared through thermal treatment or chemical imidization [26,27,28]. For copolymer systems, we chose a segment ratio of 1:1 (m:n = 1:1) as a baseline to systematically compare the effects of different structural motifs. Future studies will investigate the influence of varying segment ratios. PI-PUA copolymers chains are constructed in Figure 2.

It has been well-documented that simulation results converge to experimental values when the number of atoms in the unit cell reaches approximately 20,000 [29]. Relevant computational studies have shown that when polymerization > 10, the influence of chain ends on bulk properties becomes negligible [30]. The degree of polymerization was set to 16 for homopolymers and 21 for copolymers, thus ensuring that the total number of atoms in the all-atom models is approximately 20,000. Systems A/B/C/D/E/F consist of 30 amino-terminated molecular chains, each comprising 16 diamine molecules and 15 dianhydride or diisocyanate molecules. Systems G/H/I contain 25 molecular chains with a segment ratio m:n = 1:1 (Figure 2), composed of 21 diamine molecules, 10 dianhydride molecules, and 10 diisocyanate molecules. Systems J/K/L contain 20 molecular chains with an identical segment ratio of m:n = 1:1 (Figure 2), also consisting of 21 diamine molecules, 10 dianhydride molecules, and 10 diisocyanate molecules. The 12 model systems include 4 PI homopolymers (A/B/C/K), 4 PUA homopolymers (D/E/F/L) and 4 PI-PUA copolymers (G/H/I/J) with systematically varied chemical structures.

Monomer structures were built and geometrically optimized using the Visualizer module. Polymer chains were constructed using the Build Polymer module with the specified degree of polymerization. Amorphous cells were built using the Amorphous Cell module with an initial density of 0.2 g/cm^3^ [31,32]. Geometry optimization was first conducted using the smart minimizer algorithm, with convergence criteria set to an energy tolerance of 2 × 10^−5^ kcal/mol, a force tolerance of 0.001 kcal/mol/Å, and a displacement tolerance of 1 × 10^−5^ Å. All molecular dynamics simulations were performed using the Forcite module within the Materials Studio software suite. The COMPASS II force field was employed for all calculations, as it has been extensively validated for a wide range of organic polymer systems [15,33,34,35]. The non-bonded interaction cutoff was set to 12.5 Å, which is the recommended value for the COMPASS II force field and has been widely validated in polymer simulation studies [36], and long-range electrostatic interactions were treated using the particle-mesh Ewald (PME) summation method. The velocity Verlet algorithm was adopted as the velocity integrator with a simulation time step of 1 fs; the Andersen thermostat with a collision frequency of 1.0 ps^−1^ and the Berendsen barostat with a relaxation time constant of 0.1 ps were utilized to control temperature and pressure, respectively [37]. Three initial unit cells generated by the Amorphous Cell module were first subjected to 200 ps of NVT dynamic equilibration, followed by 2000 ps of NPT dynamic equilibration. Finally, an additional 2000 ps of dynamic relaxation was performed in the NVT ensemble, and the last 400 frames of the equilibrated system were extracted for property calculation. All calculated properties were averaged across the three model systems. The system was considered equilibrated when the total energy and density fluctuated within ±1% of their average values over the last 400 frames. The average total potential energy, total kinetic energy, and density of each equilibrated system with standard deviations are summarized in Supporting Information Table S1. All simulations were carried out with ultra-fine calculation precision to ensure numerical accuracy at 300 K and 1 atm. All property values are reported as the average of three independent simulations with different initial conditions. Error bars represent the standard deviation of the three simulations. The equilibrated model parameters are summarized in Table 1.

## 3. Results

A hierarchical, progressive grouping strategy combining single-variable control and cross-system comparison was employed to systematically analyze the 12 model systems. The systems were divided into five logically interconnected modules:

1. Systems A/B/C: Investigate the structure–property relationships of benzene rings, fluorine substituents, and cyclohexane rings in PI homopolymers;
2. Systems D/E/F: Analyze the modulatory effects of benzene rings, naphthalene rings, and aliphatic chains on PUA homopolymer properties;
3. Systems G/H/I: Elucidate the underlying action mechanisms of the aforementioned three structural motifs in PI-PUA copolymer systems;
4. Systems B/D/G, B/E/H, B/F/I: Perform horizontal comparisons of the modification effects of identical characteristic groups in three distinct polymer matrices using a “same group-different matrix” pairing approach;
5. Systems B/K, D/L, G/J: Explore the cross-system modification rules of phthalide side groups in PI, PUA, and PI-PUA systems.

This experimental design strictly adheres to the control variable principle, enabling the elucidation of synergistic regulatory mechanisms between chemical group structure and matrix composition on polymer properties through vertical single-variable investigations and horizontal cross-system validation.

### 3.1. Glass Transition Temperature

The glass transition phenomenon is characterized by abrupt changes in a wide range of physical properties, including specific volume, thermal conductivity, mechanical modulus, and dielectric constant. In this study, glass transition temperatures (*T_g_*) values were determined via bilinear fitting of density–temperature curves using Origin 2023 software, a well-established method in molecular dynamics simulations [38,39,40]. Densities corresponding to the temperatures and fitting curves (red line) of all systems are shown in the Supporting Information Figures S1–S12.

Annealing simulations were performed in the isothermal-isobaric (NPT) ensemble at a cooling rate of 25 K per 100 ps, which is approximately 12 orders of magnitude faster than typical experimental cooling rates. To account for this discrepancy, the Williams–Landel–Ferry (WLF) equation with parameters C_1_ = 17.44 and C_2_ = 51.6 [41,42,43] was employed to correct the simulated *T_g_* values. It should be noted that WLF parameters are intrinsically polymer-specific. The universal parameters used here provide a reasonable first-order correction, and polymer-specific parameters from future experiments will further improve prediction accuracy. The correction procedure revealed that simulated *T_g_* values are systematically overestimated by approximately 21.5 K relative to experimental measurements. The corrected *T_g_* values for all systems are presented in Table 2. The WLF correction is performed based on the universal WLF parameters and the fixed cooling rate ratio between molecular dynamics simulations and experimental measurements. The correction term is a constant independent of the intrinsic glass transition temperature of each system. In accordance with the principle of error propagation, a constant offset only shifts the numerical baseline without affecting the statistical fluctuation of the results. Therefore, the standard deviations of the corrected *T_g_* values are identical to those of the simulated *T_g_* values. For the sake of conciseness, the standard deviations of the corrected *T_g_* are not redundantly presented in Table 2.

To the best of our knowledge, experimental *T_g_* measurements for the full set of investigated systems have not been reported in the open literature. Nevertheless, our corrected simulation *T_g_* values are in excellent quantitative agreement with the experimental measurements previously reported for systems A and D, with a relative deviation of only 2.75% and 1.07% for system A and D. Furthermore, all systems studied herein consist solely of C, N, O, S, and F atoms, for which the COMPASS II force field provides well-validated, high-precision atomic parameters. Collectively, the good agreement between corrected *T_g_* and experimental values for systems A and D suggests that the computational method used in this work provides reasonable prediction accuracy for the thermal properties of PI and PUA systems [44,45].

**Table 2:** *T_g_* of twelve homopolymers and copolymer systems.

Crosslinking System	Simulation (K)	Correction (K)	Experiment (K)
A (DDS + PMDA)	622.56 ± 7.16	601.06	585 [46]
B (DDS + DBDI)	587.03 ± 10.13	565.53	-
C (DDS + DHTDI)	597.27 ± 9.46	575.77	-
D (DDS + PDI)	668.55 ± 6.32	647.05	654 [47]
E (DDS + HDI)	582.45 ± 3.92	560.95	-
F (DDS + NDI)	674.78 ± 10.41	653.28	-
G (DDS + DBDI + PDI)	639.90 ± 6.04	618.40	-
H (DDS + DBDI + HDI)	625.00 ± 4.81	603.50	-
I (DDS + DBDI + NDI)	643.81 ± 7.56	622.31	-
J (BAPP + DBDI + PDI)	587.36 ± 6.23	565.86	-
K (BAPP + DBDI)	615.99 ± 7.85	594.49	-
L (BAPP + PDI)	600.34 ± 3.77	578.84	-

For the PI homopolymer systems (A/B/C), the corrected *T_g_* values follow the order A (601.06 K) > C (575.77 K) > B (565.53 K). The fully aromatic backbone of PMDA imparts the highest chain rigidity, which directly translates to the highest *T_g_* observed in system A. The non-planar aliphatic ring structure of DHTDI reduces chain rigidity relative to PMDA, facilitating segmental motion and resulting in a lower *T_g_*. Despite the high electronegativity of fluorine atoms in DBDI, their incorporation increases interchain distances and fractional free volume (as shown in Section 3.3), thereby weakening intermolecular interactions and lowering the activation energy for segmental motion. This effect accounts for the lowest *T_g_* observed among the three PI homopolymer systems [21].

For the PUA homopolymer systems (D/E/F), the corrected *T_g_* values follow the order F (653.28 K) > D (647.05 K) > E (560.95 K). The naphthalene ring in NDI is a fused bicyclic aromatic moiety with exceptional rigidity and planarity, which severely restricts segmental mobility and results in the highest *T_g_* among all systems investigated. The benzene ring in PDI imparts moderate chain rigidity, leading to the intermediate *T_g_* observed in system D. In contrast, the aliphatic hexamethylene chain in HDI exhibits high conformational flexibility, significantly reducing the activation energy barrier for segmental motion. These observations are consistent with the well-established principle that aromatic isocyanates enhance the thermal stability of polyurea materials.

For the PI-PUA copolymer systems (G/H/I), the corrected *T_g_* values follow the order I (622.31 K) > G (618.40 K) > H (603.50 K), which mirrors the trend observed in the PUA homopolymer systems. Notably, the *T_g_* values of the PI-PUA copolymers are generally elevated relative to the corresponding PI homopolymers. While system G (618.40 K) exhibits a slightly lower *T_g_* than PUA homopolymer system D (647.05 K), it is substantially higher than all PI homopolymer systems. This indicates that the copolymer architecture yields *T_g_* values that are intermediate between those of pure PI and pure PUA. The positive correlation between the rigidity of the isocyanate component and *T_g_* observed in PUA homopolymers also holds true for the copolymer systems.

A cross-comparison of PI, PUA, and PI-PUA systems (B/D/G, B/E/H, B/F/I) reveals that when the PUA component incorporates rigid aromatic isocyanates (PDI, NDI), the *T_g_* of pure PUA is higher than that of pure PI, and the *T_g_* of the resulting PI-PUA copolymers falls between the two. However, when the PUA component contains the aliphatic isocyanate HDI, the *T_g_* of the copolymer system H (603.50 K) is higher than both PI homopolymer system B (565.53 K) and PUA homopolymer system E (560.95 K), indicating a clear synergistic effect. This synergistic enhancement in *T_g_* can be attributed to the formation of additional intermolecular interactions between the PI and PUA chains, which further restrict molecular motion.

The influence of phthalide side groups on *T_g_* exhibits significant system dependence. In PI homopolymers, the phthalide-containing system K (594.49 K) displays a higher *T_g_* than system B (565.53 K). In stark contrast, in both PUA homopolymers and PI-PUA copolymers, phthalide incorporation leads to a reduction in *T_g_*: system L (578.84 K) has a lower *T_g_* than system D (647.05 K), and system J (565.86 K) has a lower *T_g_* than system G (618.40 K). As a sterically bulky side group, phthalide increases the rotational steric hindrance of individual polymer chains but also significantly expands interchain distances and increases fractional free volume, thereby weakening intermolecular cohesion. The net effect of these competing factors is an overall reduction in *T_g_* upon phthalide incorporation in most systems.

### 3.2. Mechanical Properties

**Figure 3:**
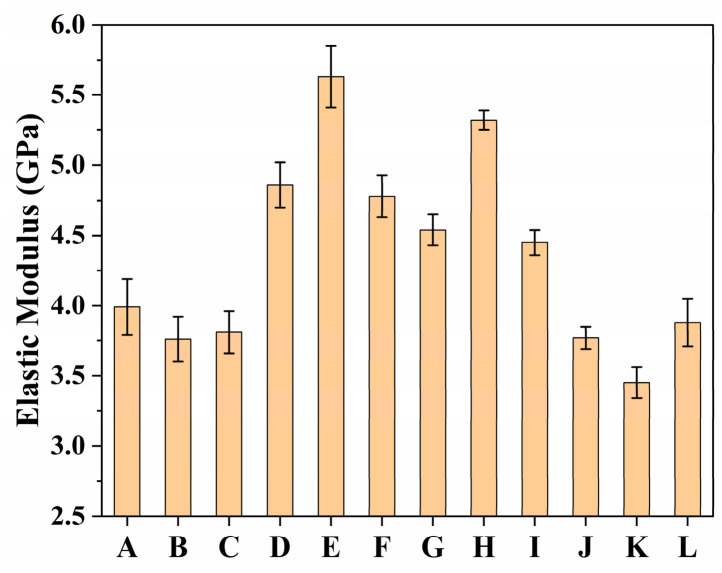
Elastic modulus of twelve systems with different monomer formulations.

**Figure 4:**
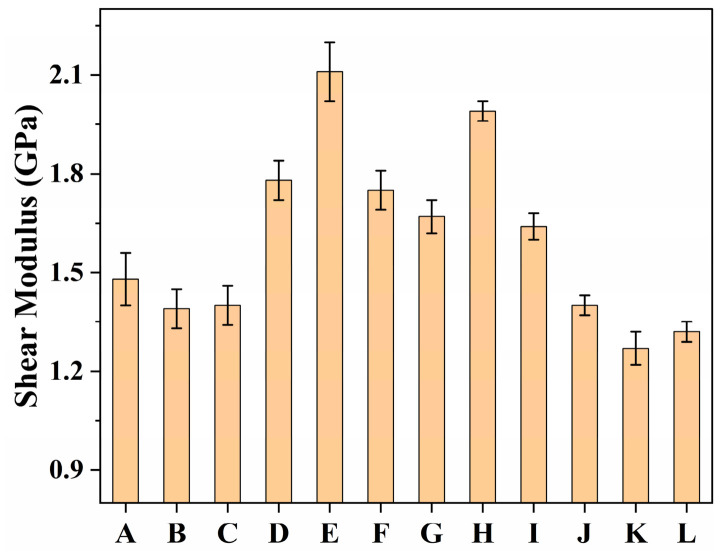
Shear modulus of twelve systems with different monomer formulations.

**Figure 5:**
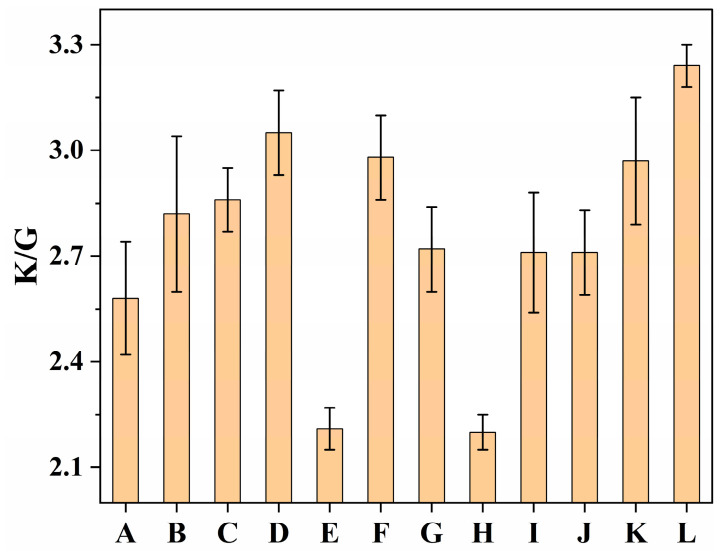
*K/G* of twelve systems with different monomer formulations.

Mechanical properties are critical determinants of the engineering applicability of polymeric materials. In this work, mechanical properties were calculated using the constant strain method, which involves applying a small uniaxial strain of 2% to the fully equilibrated systems. The elastic modulus (*E*) and shear modulus (*G*) for all systems are presented in Figure 3 and Figure 4, respectively. The ratio of bulk modulus to shear modulus (*K/G*) was employed as a quantitative metric for material toughness, where higher *K/G* values correspond to enhanced plastic deformability and thus improved toughness [48]. It should be emphasized that the K/G ratio is only a rough metric for toughness screening. Direct calculation of fracture toughness requires large-deformation simulations such as uniaxial tensile testing to failure, which will be the subject of future work. The calculated *K/G* ratios for all systems are shown in Figure 5. The calculation methods for mechanical property parameters are detailed in Supporting Information Section S2.

For the three PI homopolymer systems (A, B, and C), System A, based on PMDA with its fused benzene rings, confers the highest rigidity to the molecular chains, as evidenced by an elastic modulus of 3.99 GPa. However, the stiff aromatic rings simultaneously restrict segmental motion, leading to the lowest K/G of 2.58. In System B, the introduction of two fluorine atoms on the benzene ring of DBDI reduces the elastic and shear moduli by approximately 5.76% and 6.08%, respectively, relative to System A, whereas the K/G ratio increases by 9.30%. Owing to the intrinsically low atomic polarizability and strong electron-withdrawing character of fluorine, fluorine-containing moieties reduce the molecular polarizability and dielectric constant of the polymer systems, enhance their hydrophobicity, high-frequency dielectric loss performance, and optical bandgap, and concurrently improve chain flexibility [49,50]. In System C, replacing the benzene ring of PMDA with the cyclohexane alicyclic structure of DHTDI leads to reductions in elastic and shear moduli of 4.51% and 5.41%, respectively, and an increase in the K/G ratio of 10.85%, confirming that the cyclohexane moiety enhances toughness by increasing chain flexibility while preserving relatively high rigidity [51].

For the PUA homopolymer systems (D/E/F), system E, which contains aliphatic hexamethylene chains, exhibits the highest moduli but the lowest toughness among all PUA systems. In contrast, the aromatic isocyanate-based systems F and D display slightly lower moduli but significantly enhanced toughness. This inverse relationship between modulus and toughness can be rationalized by fundamental differences in hydrogen bond network topology and its effect on energy dissipation mechanisms. In system E, the flexible aliphatic hexamethylene chains of HDI enable the formation of a dense, spatially homogeneous hydrogen bond network, wherein hydrogen bonds act as rigid physical crosslinks that strongly restrict segmental mobility. While this dense network enhances elastic modulus through increased intermolecular cohesion, it simultaneously suppresses interchain slippage and eliminates hierarchical energy dissipation pathways. Consequently, when external stress is applied, stress concentration cannot be effectively relieved through sequential bond breakage, leading to brittle fracture with limited plastic deformation [52]. In contrast, the aromatic isocyanate-based systems D (PDI) and F (NDI) possess rigid aromatic rings that introduce steric constraints, disrupting the spatial uniformity of hydrogen bond formation and creating a heterogeneous network with both strong and weak hydrogen bonds. This heterogeneity enables hierarchical energy dissipation: weaker hydrogen bonds break first under deformation, absorbing energy and allowing chain reorientation, while stronger bonds maintain structural integrity [53]. This mechanism explains why systems D and F exhibit lower moduli but significantly enhanced toughness (K/G = 3.05 and 2.98, respectively) compared to system E.

For the PI-PUA copolymer systems (G/H/I), the mechanical property values of the PI-PUA copolymer systems vary in a manner consistent with that observed for the PUA homopolymers, indicating that the mechanical properties of the copolymers are primarily governed by the PUA component.

A cross-comparison of all systems reveals that the mechanical properties of the PI-PUA copolymers are intermediate between those of pure PI and pure PUA homopolymers. When the copolymer incorporates aliphatic HDI chains, system H achieves a modulus comparable to that of PUA homopolymer system E but with significantly improved toughness. For copolymers containing aromatic isocyanates (PDI, NDI), systems G and I exhibit toughness levels comparable to those of PI homopolymer system B while maintaining high mechanical stiffness. These results collectively confirm that PI-PUA copolymerization is an effective strategy to achieve a balanced combination of rigidity and toughness in polymeric materials.

**Figure 6:**
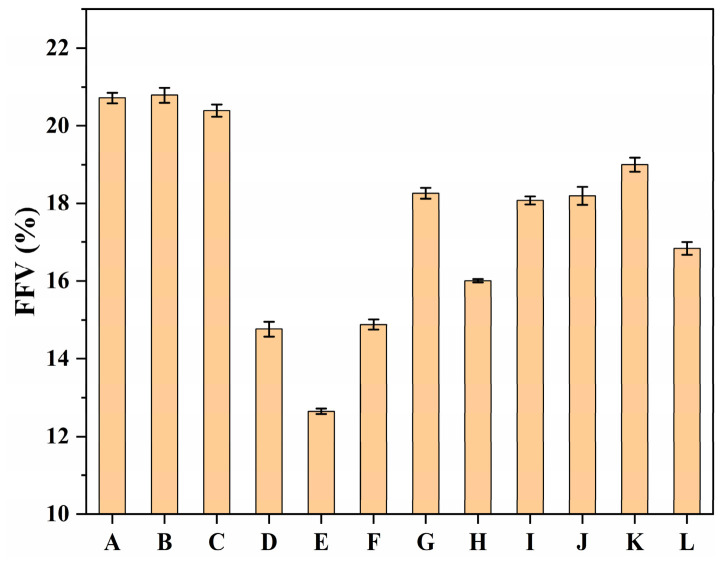
FFV of twelve systems with different monomer formulations.

The phthalide-containing systems (J/K/L) generally exhibit high K/G ratios. System L achieves the highest K/G value of 3.24, which as a preliminary screening indicator suggests its superior plastic deformation potential. As a sterically bulky side group, phthalide effectively expands interchain distances and increases fractional free volume (as shown in Figure 6, labels D and L), providing sufficient free space for conformational rearrangement and interchain slippage under shear stress. This effect significantly enhances the plastic deformation capacity of the material, resulting in the exceptional toughness observed in phthalide-containing systems.

### 3.3. Fraction Free Volume

Fractional free volume (FFV) is a direct reflection of the packing density of molecular chains and represents a key microstructural parameter governing multiple critical polymer properties. FFV was calculated using the Connolly surface method, which is the most widely used technique for determining free volume in amorphous polymers [54]. This method calculates the volume accessible to a hard-sphere probe as it rolls over the van der Waals surface of the molecules. A spherical probe with a radius of 0.1 nm was used to move along the van der Waals surface to generate a Connolly surface. The volume between the probe atom and the van der Waals surface is considered free volume [55]. The grid interval and van der Waals (vdW) scale factor were set to 0.2 and 1, respectively. The calculated FFV results for all systems are presented in Figure 6.

For the PI homopolymer systems (A/B/C), the FFV of the fluorine-containing system B is slightly higher than that of system A, but the difference is within statistical uncertainty. This indicates that fluorine substitution has a negligible effect on FFV in PI homopolymer systems. Cyclohexane-containing system C shows lower FFV and higher first peak in the intermolecular RDF (Supporting Information Figure S13) than benzene-containing systems A/B, indicating more compact chain packing in this specific PI system. This phenomenon can be attributed to the chair conformation of cyclohexane, which enables tighter interlocking of adjacent chains.

For the PUA homopolymer systems (D/E/F), the aromatic isocyanate-based systems F and D exhibit comparable FFV values that are substantially higher than that of the aliphatic isocyanate-based system E. This observation can be rationalized by the fact that flexible aliphatic chains possess higher conformational flexibility, enabling them to pack more densely and thus reduce fractional free volume.

For the PI-PUA copolymer systems (G/H/I), the FFV values of the PI-PUA copolymer systems vary in a manner consistent with that observed for the PUA homopolymers, implying that the isocyanate moiety plays a dominant role in controlling the free volume.

A cross-comparison of all 12 systems reveals that the FFV values of the copolymer systems are intermediate between those of pure PI and pure PUA homopolymers. This suggests that the incorporation of PUA components reduces the overall free volume of the systems and enhances molecular chain packing density, which correlates well with the improved mechanical moduli observed in the copolymer systems.

The influence of phthalide side groups on FFV exhibits strong matrix dependence. In PI homopolymers, the phthalide-containing system K (19.00%) displays a lower FFV than system B (20.79%), indicating that phthalide groups promote more efficient chain packing in PI matrices. In contrast, in PUA homopolymers, the phthalide-containing system L (16.83%) exhibits a higher FFV than system D (14.76%), suggesting that phthalide groups disrupt chain regularity and increase free volume in PUA matrices. In PI-PUA copolymers, the FFV of the phthalide-containing system J (18.19%) is nearly identical to that of system G (18.26%), indicating that phthalide incorporation has a negligible effect on free volume in this copolymer matrix.

### 3.4. Cohesion Energy Density

**Figure 7:**
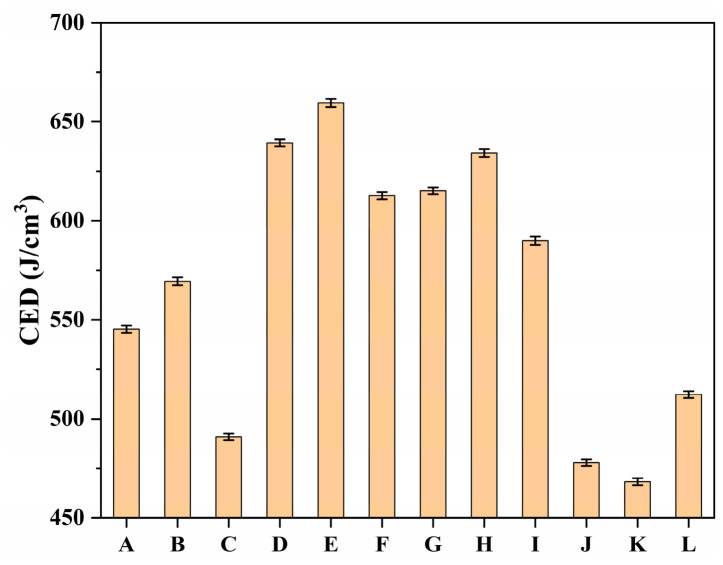
CED of twelve systems with different monomer formulations.

CED is a fundamental parameter that quantifies the strength of intermolecular interactions within a material, which directly governs its mechanical properties and solubility characteristics. CED was computed as the difference between the total non-bonded interaction energy and the intramolecular energy, normalized by the volume of the equilibrated system. The calculated CED values for all systems are presented in Figure 7.

For the PI homopolymer systems (A/B/C), the CED values follow the order B (569.40 J/cm^3^) > A (545.29 J/cm^3^) > C (490.89 J/cm^3^). The highest CED observed in the fluorine-substituted system B initially appears counterintuitive, as fluorine incorporation is generally expected to weaken intermolecular interactions. For highly symmetric structures (PMDA and DBDI), the intrinsic theoretical dipole moment is strictly zero. The dipole moment of the monomer was calculated using the hybrid B3LYP functional in the DMol3 module of Materials Studio. A double numerical plus polarization (DNP) was set as the basis set for the computations. This model is the same as the 6-31G** model in Gaussian computational software [56,57,58]. The core in the DFT-D simulations was established using the semi-core pseudopods approach, while the orbital cut-off was adjusted to 5 Å for each atom during the DFT-D computations. The maximum force was adjusted to 0.004 H/Å. The long-range dispersion force was corrected using the Grimme method [59]. The theoretically computed dipole moments of PMDA and DBDI are 0.001 debye and 0.0006 debye, respectively, which are broadly consistent with the PMDA dipole moment values (0.002 debye and 0.0011 debye) reported by Chung [60] and Fonari [61]; such minor discrepancies arise from computational floating-point errors. The dipole moments of DDS-PMDA-DDS and DDS-DBDI-DDS molecules were calculated to be 3.5975 debye and 11.3080 debye, respectively. This result verifies the strong polarization effect induced by the two fluorine substituents on the benzene ring of DBDI, which strengthens the local dipole–dipole interactions and thereby increases the overall cohesive energy of the system. Conversely, the lowest CED observed in the cyclohexane-containing system C indicates that non-polar cyclohexane moieties result in weaker intermolecular interactions.

For the PUA homopolymer systems (D/E/F), the CED values follow the order E (659.50 J/cm^3^) > D (639.40 J/cm^3^) > F (612.80 J/cm^3^). The highest CED observed in the aliphatic isocyanate-based system E is closely correlated with the hydrogen bond density in PUA systems. As previously reported, aliphatic isocyanates tend to form more extensive hydrogen bond networks, leading to stronger intermolecular interactions and higher cohesive energy [62].

For the PI-PUA copolymer systems (G/H/I), the CED values follow the order H (634.15 J/cm^3^) > G (615.21 J/cm^3^) > I (589.90 J/cm^3^), which is consistent with the trend observed in the PUA homopolymer systems. This confirms that hydrogen bonding remains the dominant intermolecular interaction in the PI-PUA copolymer systems.

A cross-comparison of all systems demonstrates that the CED values of the copolymer systems are intermediate between those of pure PI and pure PUA homopolymers. This indicates that the incorporation of PUA components enhances the overall intermolecular interactions in PI-PUA systems, which is a key factor contributing to the improved mechanical moduli of the copolymers.

The incorporation of phthalide side groups generally reduces the CED of the systems: the CED values of phthalide-containing systems K (468.26 J/cm^3^), L (512.20 J/cm^3^), and J (477.80 J/cm^3^) are all lower than those of their corresponding reference systems B, D, and G, respectively. This reduction in CED can be attributed to the steric hindrance imposed by the bulky phthalide side groups, which disrupt close intermolecular contacts and weakens intermolecular interactions, thus lowering the cohesive energy density.

### 3.5. Hydrogen Bond

**Figure 8:**
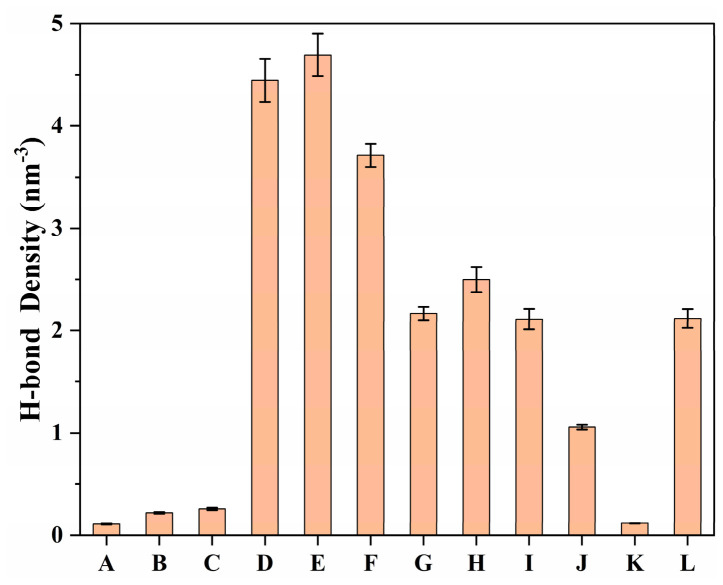
H-bond Density of twelve systems with different monomer formulations.

Hydrogen bonding plays a pivotal role in determining the mechanical and thermal properties of polymeric materials and represents the dominant intermolecular interaction governing the performance of PUA and PI-PUA copolymer systems. Hydrogen bond density (HBD), defined as the number of hydrogen bonds per unit volume (nm^−3^), is a critical parameter that characterizes the structural integrity of the internal hydrogen bond network and exerts a profound influence on material stability [63]. In this study, a hydrogen bond was considered to exist when the distance between the hydrogen donor and acceptor was less than 3.5 Å and the donor-hydrogen-acceptor angle was greater than 130° [64]. The number of hydrogen bonds in each system was counted using a custom Perl script, and the calculated HBD values for all systems are presented in Figure 8.

In the PI homopolymer systems (A/B/C), the HBD values are extremely low (<0.26 nm^−3^). This is because PIs are primarily held together by van der Waals forces and dipole–dipole interactions, with only a negligible number of hydrogen bonds present in the structure.

For the PUA homopolymer systems (D/E/F), the HBD values follow the order E (4.70 nm^−3^) > D (4.45 nm^−3^) ≈ F (3.71 nm^−3^). The aliphatic isocyanate-based system E exhibits comparable and the highest HBD values, while the naphthalene isocyanate-based system D displays a slightly lower HBD, and system F shows the lowest HBD value. This trend is in excellent agreement with the CED results, further confirming that hydrogen bonding is the dominant intermolecular interaction in PUA systems.

For the PI-PUA copolymer systems (G/H/I), the HBD values follow the order H (2.50 nm^−3^) > G (2.17 nm^−3^) ≈ I (2.11 nm^−3^). This indicates that the PUA component remains the primary source of hydrogen bonds in the copolymer systems.

A cross-comparison of all systems reveals that the HBD values of the copolymer systems are intermediate between those of pure PI and pure PUA homopolymers. Specifically, the HBD values of the PI-PUA systems (G/H/I, 2.11–2.50 nm^−3^) are approximately half those of the pure PUA systems (D/E/F, 3.71–4.70 nm^−3^), which correlates well with the reduced molar fraction of polyurea segments in the copolymer structures.

The incorporation of phthalide side groups significantly reduces the HBD of the systems: the HBD of the phthalide-containing system K is marginally lower than that of system B, while the HBD values of the phthalide-containing systems L (2.12 nm^−3^) and J (1.06 nm^−3^) are substantially lower than those of their respective reference systems D and G. This reduction in HBD can be attributed to the steric hindrance effect of the bulky phthalide side groups, which physically impedes the formation of hydrogen bonds between adjacent urea groups, thus decreasing the overall hydrogen bond density.

To further elucidate the thermal stability of the hydrogen bond network in each system, the temperature dependence of HBD was investigated over the temperature range of 300 K to 600 K. The results of this systematic analysis are summarized in Table 3.

**Figure 9:**
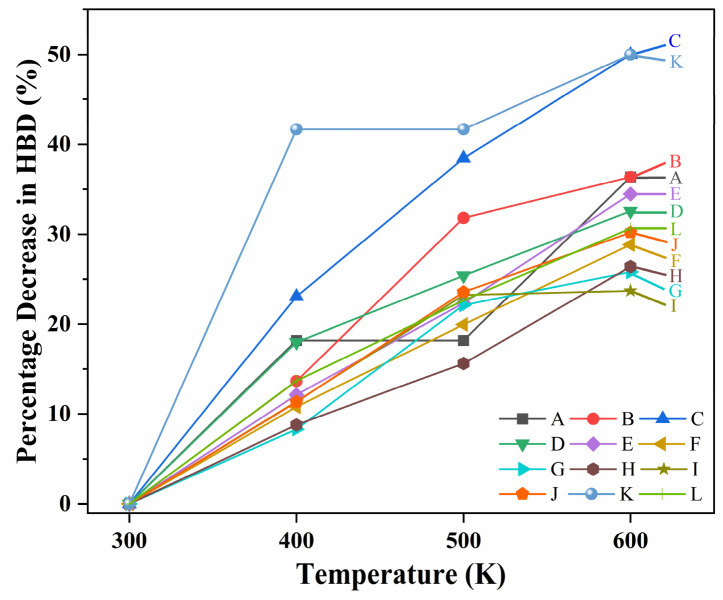
Temperature dependence of the percentage decrease in HBD for twelve systems over the temperature range of 300–600 K.

As clearly demonstrated in Table 3, the HBD values of all systems decrease monotonically with increasing temperature, which is consistent with the well-established principle that intensified thermal motion promotes hydrogen bond dissociation. However, substantial differences in the rate of HBD decay are observed among different systems, reflecting fundamental differences in the thermal stability of their respective hydrogen bond networks. The percentage decrease in HBD compared with 300 K are presented in Figure 9.

The PI homopolymer systems (A/B/C) exhibit extremely low HBD values with weak temperature dependence. Due to the absence of urea linkages, PI systems (A/B/C) can only form weak hydrogen bonds between the carbonyl groups in the imide rings and a small number of N-H groups in the diamine residues, resulting in HBD values of only 0.11–0.26 nm^−3^ at 300 K. The temperature dependence of this weak hydrogen bond network is also minimal, with only a limited reduction in HBD observed over the temperature range from 300 K to 600 K. This reflects the fact that van der Waals forces and π-π stacking interactions are the dominant intermolecular interactions in PI systems.

The PUA homopolymer systems (D/E/F) exhibit the highest HBD values but show significant differences in hydrogen bond network thermal stability. At 300 K, the HBD values of the PUA systems range from 3.71 to 4.70 nm^−3^, which is substantially higher than those of the PI systems (A/B/C, 0.11–0.26 nm^−3^) and PI-PUA copolymer systems (G/H/I, 2.11–2.50 nm^−3^). Notably, system E maintains the highest HBD across the entire temperature range investigated, decreasing from 4.70 to 3.08 nm^−3^, which corresponds to a reduction of only 34.47%. This exceptional thermal stability originates from the high conformational flexibility of the linear aliphatic chains in HDI, which enables the molecular chains to adjust their conformations in response to thermal perturbation, thereby maintaining the optimal donor–acceptor distance for urea hydrogen bond formation.

In comparison, the HBD of system D decreases from 4.45 to 3.00 nm^−3^ (a reduction of 32.58%), while that of system F decreases from 3.71 to 2.64 nm^−3^ (a reduction of 28.84%). Aromatic isocyanate-based systems (D, F) show slightly lower HBD reduction rate than the aliphatic system E, indicating better thermal stability of their hydrogen bond networks. This is because the rigid aromatic backbone provides structural support for hydrogen bond pairs, retarding their disruption by thermal motion.

The HBD values of the PI-PUA copolymer systems (G/H/I) are intermediate between those of PI and PUA homopolymers, and their hydrogen bond network thermal stability exhibits trends similar to those of the corresponding PUA systems. System H exhibits the highest initial HBD (2.50 nm^−3^) and a relatively low reduction of 26.40% over the temperature range, which is consistent with its excellent elastic modulus of 5.36 GPa. The HBD decay amplitudes of system G (25.81%) and system I (23.70%) are comparable.

A comprehensive analysis of the temperature dependence of HBD demonstrates that PUA systems not only possess the highest HBD values but also exhibit excellent hydrogen bond network thermal stability, which is crucial for maintaining mechanical performance at elevated temperatures. However, an excessively dense and strong hydrogen bond network may inhibit interchain slippage and thus reduce material toughness, highlighting the importance of optimizing hydrogen bond density to achieve a balanced combination of mechanical properties.

## 4. Conclusions

In this work, we have employed all-atom molecular dynamics simulations to systematically investigate the thermal and mechanical properties of 12 distinct PI, PUA, and PI-PUA copolymer systems. The molecular-level regulatory mechanisms of key structural motifs on material performance have been elucidated, leading to the following principal conclusions:

1. Rigid aromatic moieties (PMDA, NDI) confer the highest *T_g_* and outstanding elastic moduli to the resulting materials, albeit with only moderate toughness. System E, which incorporates flexible aliphatic hexamethylene diisocyanate (HDI) chains, exhibits a canonical “high rigidity–low toughness” trade-off. Mechanistically, the dense, spatially homogeneous hydrogen-bond network formed by flexible HDI segments functions as rigid physical cross-linking points that elevate the elastic modulus but restrict interchain slippage and eliminate hierarchical energy-dissipation pathways, ultimately resulting in brittle fracture. In contrast, aromatic isocyanate-based systems (D, F) feature heterogeneous hydrogen-bond networks that enable sacrificial bond-mediated energy dissipation, thereby delivering substantially superior toughness.
2. The BAPP-based series (J/K/L), which incorporate phthalide side groups, exhibit superior plastic deformation capacity due to their high K/G ratios (2.71–3.24), making them potential candidate structures for further experimental investigation for applications requiring high toughness. Among these systems, system J achieves the optimal balance between rigidity and toughness, maintaining a moderate elastic modulus of 3.68 GPa while possessing the highest K/G ratio of 3.24.
3. Fluorine substitution (DBDI) and cyclohexane ring incorporation (DHTDI) have relatively minor effects on mechanical modulus but can be employed to fine-tune the low-temperature toughness and dielectric properties of polymeric materials by modulating fractional free volume and cohesive energy density.
4. The PI-PUA copolymerization strategy effectively balances the rigidity and toughness of polymeric materials across a broad performance spectrum. For instance, system G maintains a high elastic modulus while exhibiting toughness comparable to PI homopolymers, confirming that copolymerization is a viable technical approach to overcome the inherent performance limitations of single-component PI and PUA materials.

This study provides insights into the structure–property relationships of PI-PUA copolymer systems. All simulation predictions in this work require experimental validation before practical application. Future research efforts will focus on investigating the effects of copolymer segment ratio, crosslink density, and temperature on dynamic mechanical properties, as well as validating the simulation predictions through systematic experimental synthesis and characterization. Furthermore, multi-scale simulations integrating molecular dynamics and finite element analysis will be performed to establish quantitative correlations between microscopic intermolecular interactions and macroscopic material performance.

## Figures and Tables

**Table 1:** Model parameter of twelve homopolymers and copolymer systems.

System	Atom Number	Diamine	Anhydride	Isocyanate	Density (g/cm^3^)
A (DDS + PMDA)	19,740	DDS	PMDA	-	1.36
B (DDS + DBDI)	19,320	DDS	DBDI	-	1.43
C (DDS + DHTDI)	22,020	DDS	DHTDI	-	1.31
D (DDS + PDI)	21,120	DDS	-	PDI	1.35
E (DDS + HDI)	24,720	DDS	-	HDI	1.28
F (DDS + NDI)	23,820	DDS	-	NDI	1.34
G (DDS + DBDI + PDI)	22,225	DDS	DBDI	PDI	1.39
H (DDS + DBDI + HDI)	23,200	DDS	DBDI	HDI	1.36
I (DDS + DBDI + NDI)	22,700	DDS	DBDI	NDI	1.38
J (BAPP + DBDI + PDI)	22,520	BAPP	DBDI	PDI	1.29
K (BAPP + DBDI)	21,960	BAPP	DBDI	-	1.32
L (BAPP + PDI)	23,080	BAPP	-	PDI	1.26

**Table 3:** Temperature dependence of hydrogen bond density of twelve systems.

System	A	B	C	D	E	F	G	H	I	J	K	L
HBD (nm^−3^)/300 K	0.11	0.22	0.26	4.45	4.70	3.71	2.17	2.50	2.11	1.06	0.12	2.12
HBD (nm^−3^)/400 K	0.09	0.19	0.20	3.65	4.13	3.31	1.99	2.28	1.87	0.94	0.07	1.83
HBD (nm^−3^)/500 K	0.09	0.15	0.16	3.32	3.65	2.97	1.69	2.11	1.62	0.81	0.07	1.64
HBD (nm^−3^)/600 K	0.07	0.14	0.13	3.00	3.08	2.64	1.61	1.84	1.61	0.74	0.06	1.47

## Data Availability

The original contributions presented in this study are included in the article/Supplementary Material. Further inquiries can be directed to the corresponding authors.

## Supplementary Materials

The following supporting information can be downloaded at: https://www.mdpi.com/article/10.3390/polym18141779/s1.
