# Supplementary material for: Thermal and Mechanical Behavior of Polyimide–Polyurea Copolymers: Insights from Molecular Dynamics Simulations
Source: Polymers (Basel). 2026 Jul 21;18(14):1779. doi: 10.3390/polym18141779 (PMC13417153; doi:10.3390/polym18141779)
Supplement: Supplementary file 1 [file polymers-18-01779-s001.zip › polymers-4333467-supplementary.pdf]

Supporting Information for:

Thermal and Mechanical Behavior of Polyimide–Polyurea Copolymers: Insights from Molecular Dynamics Simulations

1 Glass Transition Temperature

Densities corresponding to the temperatures and fitting curves (red line) of all systems are shown in Figures S1-S12.

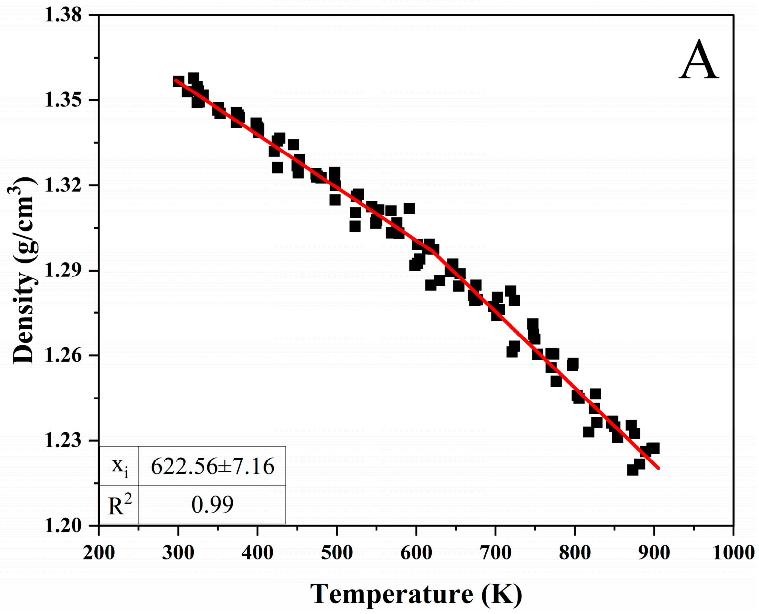

Figure S1. Densities corresponding to the temperatures and fitting curves for system A

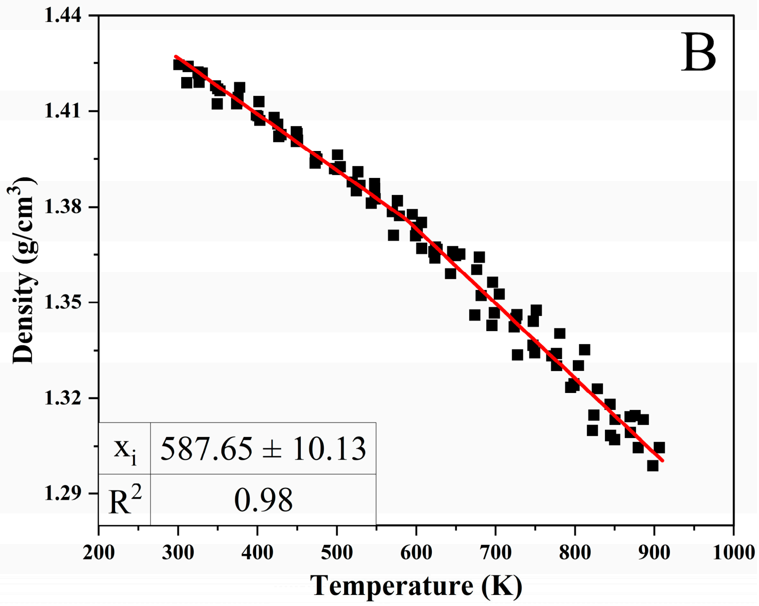

Figure S2. Densities corresponding to the temperatures and fitting curves for system B

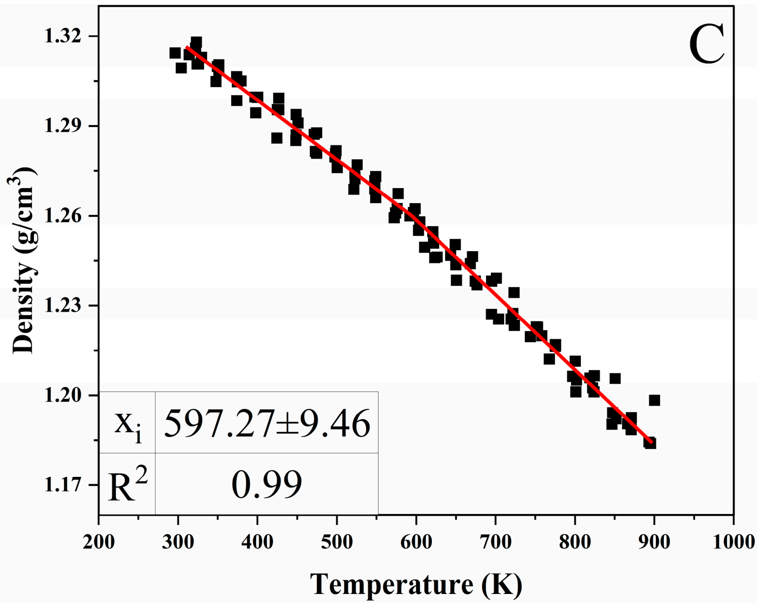

Figure S3. Densities corresponding to the temperatures and fitting curves for system C

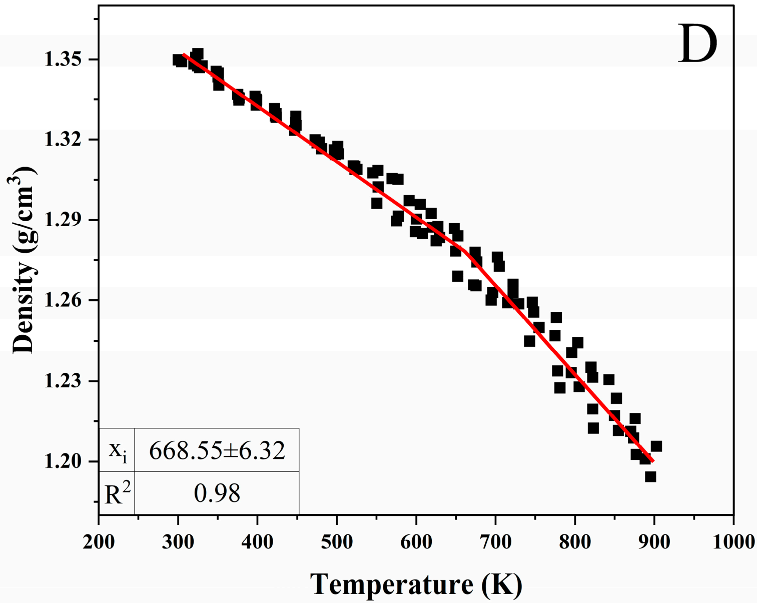

Figure S4. Densities corresponding to the temperatures and fitting curves for system D

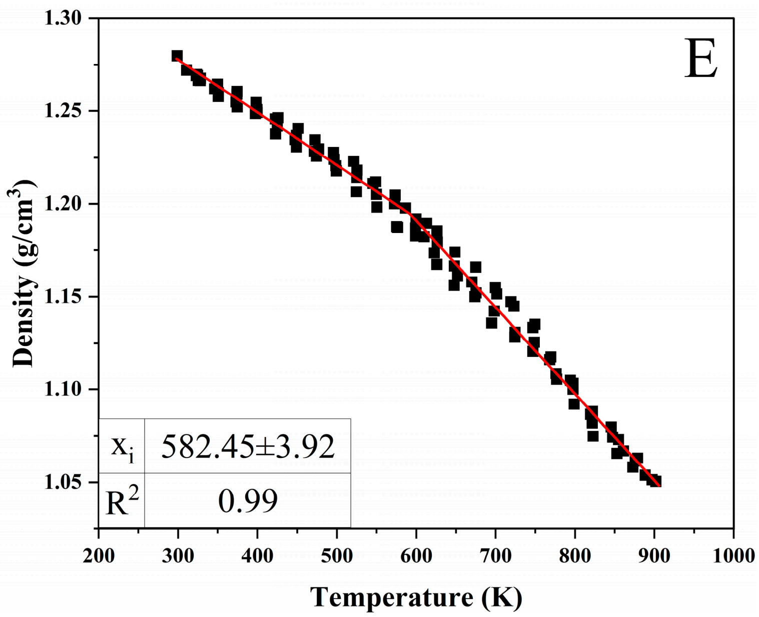

Figure S5. Densities corresponding to the temperatures and fitting curves for system E

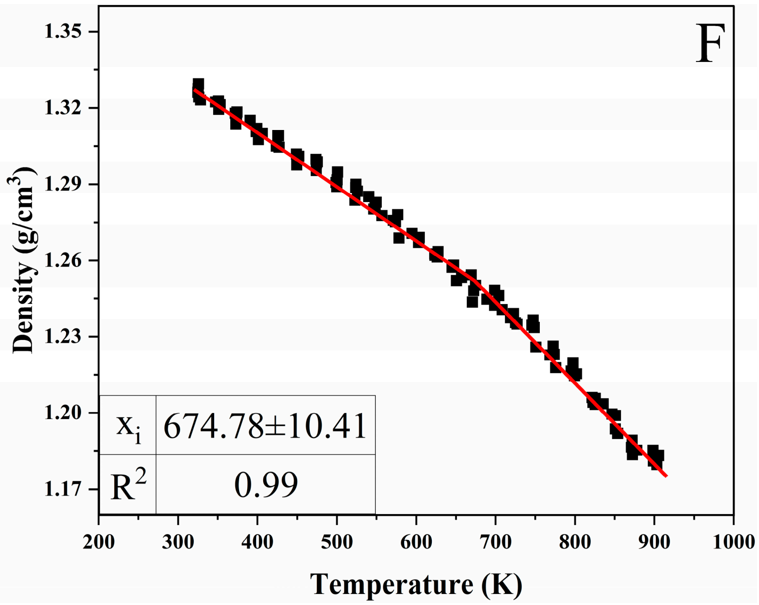

Figure S6. Densities corresponding to the temperatures and fitting curves for system F

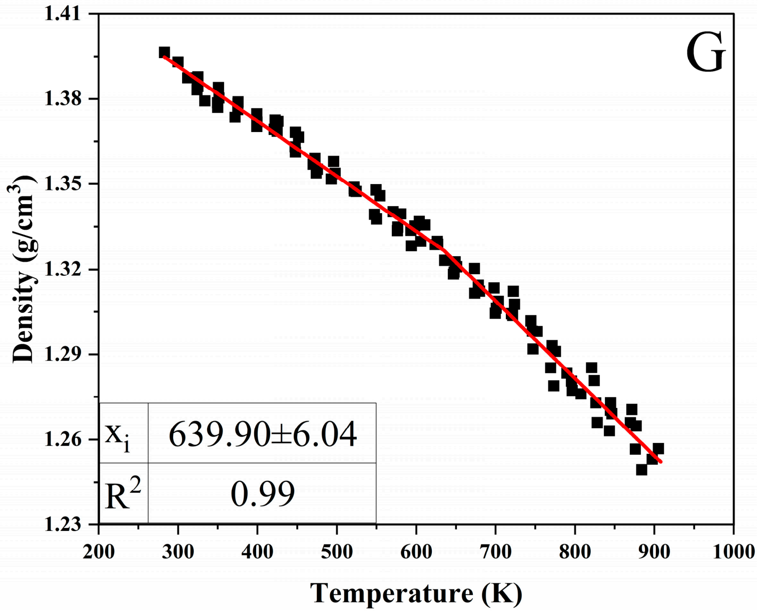

Figure S7. Densities corresponding to the temperatures and fitting curves for system G

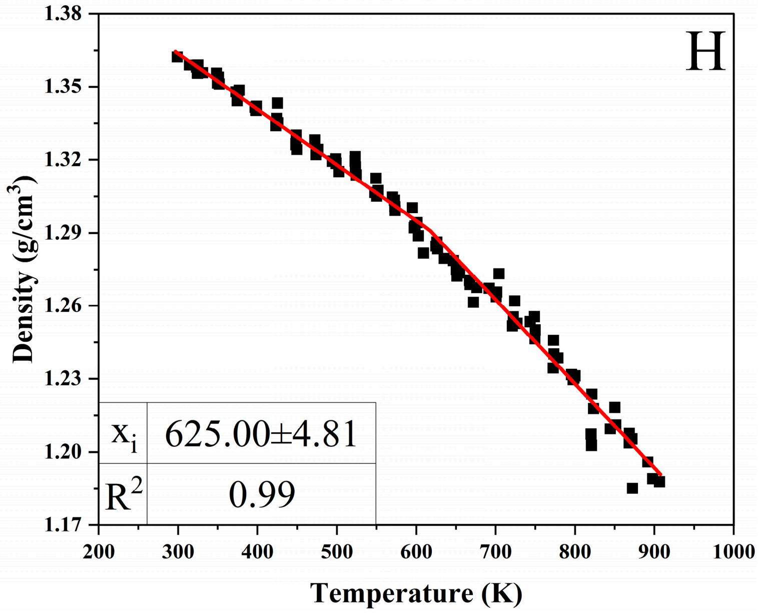

Figure S8. Densities corresponding to the temperatures and fitting curves for system H

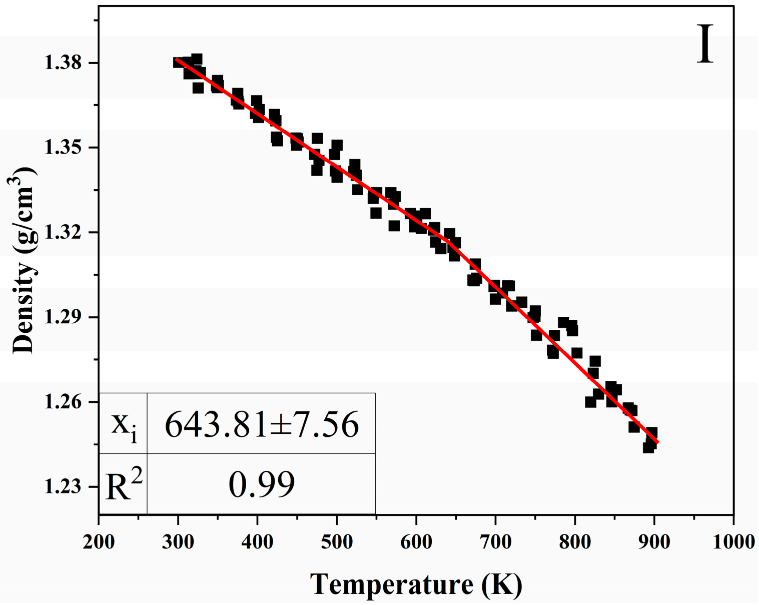

Figure S9. Densities corresponding to the temperatures and fitting curves for system I

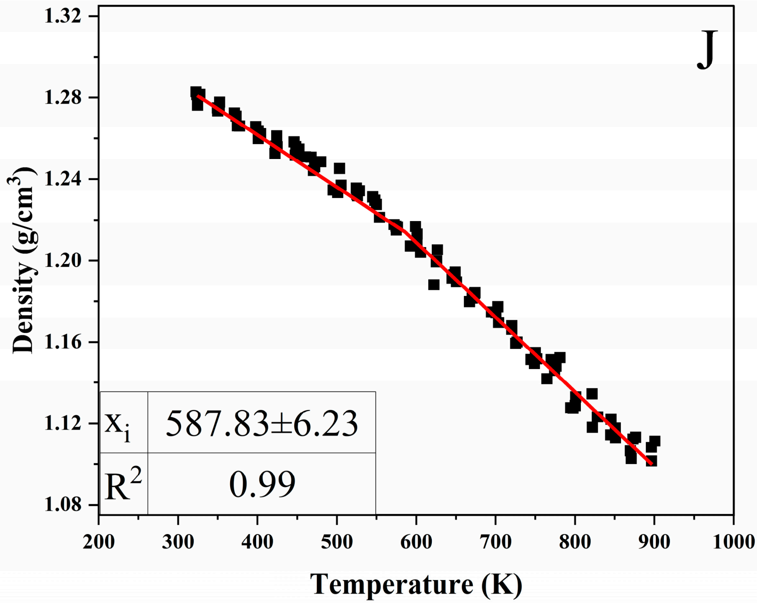

Figure S10. Densities corresponding to the temperatures and fitting curves for system J

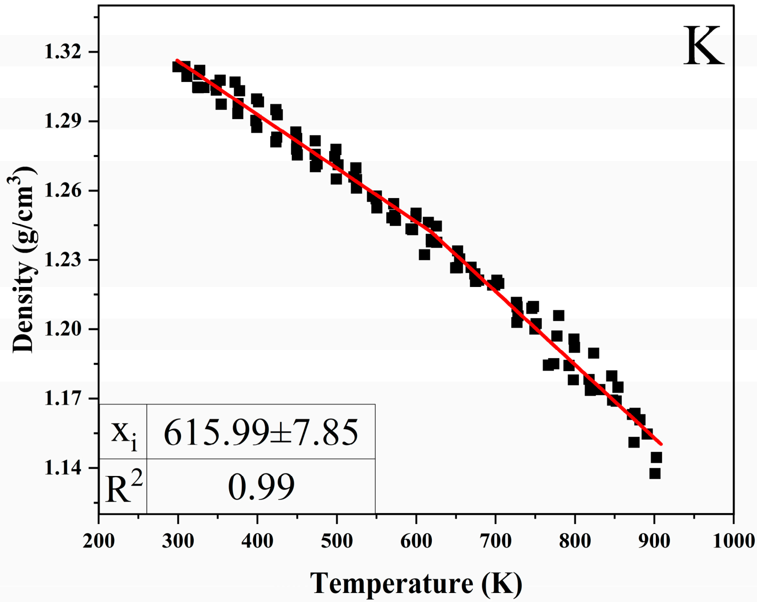

Figure S11. Densities corresponding to the temperatures and fitting curves for system K

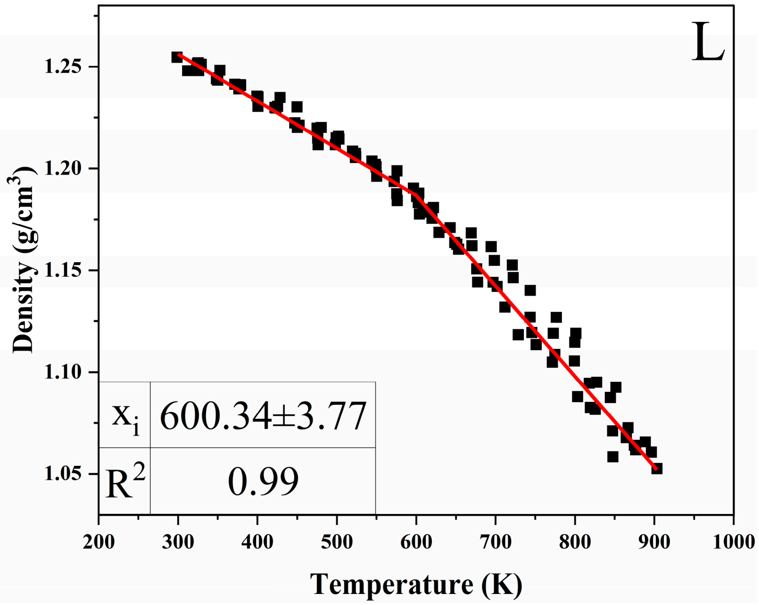

Figure S12. Densities corresponding to the temperatures and fitting curves for system L

2 Mechanical Properties

For an isotropic material, the stiffness matrix can be described by two independent coefficients. The simplified stiffness matrix can be written as follows:

$$\begin{bmatrix} C_{11} & C_{12} & C_{13} & C_{14} & C_{15} & C_{16} \\ C_{21} & C_{22} & C_{23} & C_{24} & C_{25} & C_{26} \\ C_{31} & C_{32} & C_{33} & C_{34} & C_{35} & C_{36} \\ C_{41} & C_{42} & C_{43} & C_{44} & C_{45} & C_{46} \\ C_{51} & C_{52} & C_{53} & C_{54} & C_{55} & C_{56} \\ C_{61} & C_{62} & C_{63} & C_{64} & C_{65} & C_{66} \end{bmatrix} = \begin{bmatrix} \lambda+2\mu & \lambda & \lambda & 0 & 0 & 0 \\ \lambda & \lambda+2\mu & \lambda & 0 & 0 & 0 \\ \lambda & \lambda & \lambda+2\mu & 0 & 0 & 0 \\ 0 & 0 & 0 & \mu & 0 & 0 \\ 0 & 0 & 0 & 0 & \mu & 0 \\ 0 & 0 & 0 & 0 & 0 & \mu \end{bmatrix} \tag{1}$$

$$\lambda = \frac{1}{3}\left(C_{11} + C_{22} + C_{33}\right) - \frac{2}{3}\left(C_{44} + C_{55} + C_{66}\right) \tag{2}$$

$$\mu=\frac{1}{3}(C_{44}+C_{55}+C_{66}) \tag{3}$$

where λ and μ are referred to as the Lamé coefficients in matrix 1. For the isotropic case, the elastic modulus E, bulk modulus K, and shear modulus G can be expressed in terms of the Lamé coefficients as follows:

$$E=\mu\left(\frac{3\lambda+2\mu}{\lambda+\mu}\right) \tag{4}$$

$$K=\lambda+\frac{2}{3\mu} \tag{5}$$

$$G=\mu \tag{6}$$

3 Radial Distribution Function (RDF)

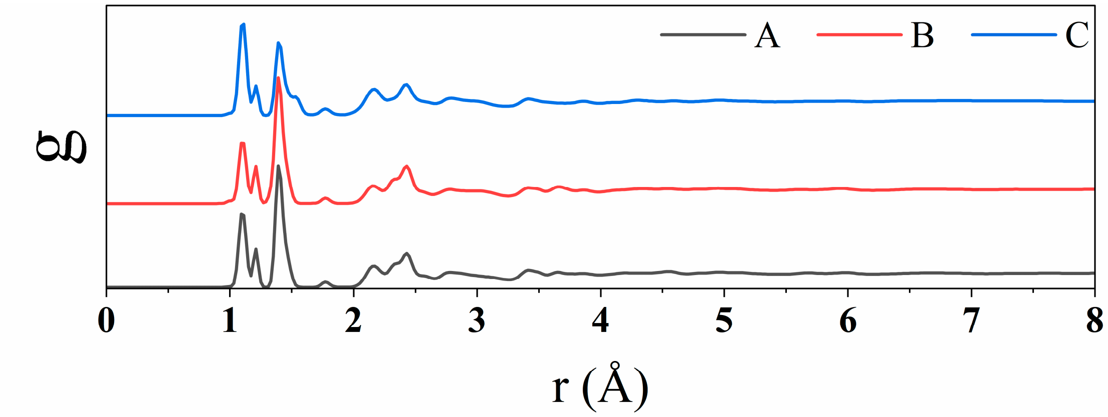

Figure S13. PI homopolymer RDF of A, B, C, systems

4 Thermodynamic characteristics

Table S1 presents the mean total potential energy, total kinetic energy, and density, along with their standard deviations, for A-L equilibrated systems. A system was considered to have reached equilibrium when the fluctuations in total energy and density over the final 400 frames remained within ±1% of their respective mean values.

Table S1. Equilibrium total potential energy, total kinetic energy, density and their relative fluctuations for A-L systems

System	potential energy	fluctuation	kinetic energy	fluctuation	density	fluctuation

A（DDS+PMDA）	13145.79±85.31	0.65%	17621.00±87.39	0.50%	1.36±0.0020	0.14%
B（DDS+DBDI）	-10540.47±85.66	0.81%	17266.53±80.20	0.46%	1.43±0.0022	0.15%
C（DDS+DHTDI）	6595.22±58.27	0.88%	19655.70±90.87	0.46%	1.31±0.0022	0.17%
D（DDS+PDI）	-70670.12±93.47	0.13%	18861.66±86.58	0.46%	1.35±0.0018	0.13%
E（DDS+HDI）	-75937.53±101.16	0.13%	22063.41±94.30	0.43%	1.28±0.0017	0.13%
F（DDS+NDI）	-13121.72±95.25	0.73%	21266.88±83.37	0.39%	1.34±0.0016	0.12%
G（DDS+DBDI+PDI）	-45251.04±87.20	0.19%	19858.01±80.84	0.41%	1.39±0.0021	0.15%
H（DDS+DBDI+HDI）	-48090.93±91.09	0.19%	20725.54±101.46	0.49%	1.36±0.0018	0.14%
I（DDS+DBDI+NDI）	-12668.74±95.26	0.75%	20262.68±97.68	0.48%	1.38±0.0021	0.15%
J（BAPP+DBDI+PDI）	-32034.83±94.76	0.30%	20116.29±90.32	0.45%	1.29±0.0020	0.15%
K（BAPP+DBDI）	-13720.47±91.25	0.67%	19623.08±87.47	0.45%	1.33±0.0021	0.16%
L（BAPP+PDI）	-49947.60±87.74	0.18%	20619.64±77.03	0.37%	1.26±0.0019	0.15%
